# Tetra­kis(1-ethyl-3-methyl­imidazolium) β-hexa­cosa­oxidoocta­molybdate

**DOI:** 10.1107/S1600536808015936

**Published:** 2008-06-21

**Authors:** Shiwei Lin, Weilin Chen, Zhiming Zhang, Wenli Liu, Enbo Wang

**Affiliations:** a Key Laboratory of Polyoxometalate Science of the Ministry of Education, Department of Chemistry, Northeast Normal University, Changchun, Jilin 130024, People’s Republic of China; b Department of Chemistry, Changchun Normal University, Changchun, Jilin 130024, People’s Republic of China

## Abstract

The title compound, (C_6_H_11_N_2_)_4_[Mo_8_O_26_] or (emim)_4_[β-Mo_8_O_26_] (emim is 1-ethyl-3-methyl­imidazolium), was obtained from the ionic liquid [emim]BF_4_. The asymmetric unit contains two [emim]^+^ cations and one-half of the [β-Mo_8_O_26_]^4−^ tetra­anion, which occupies a special position on an inversion centre. The β-[Mo_8_O_26_]^4−^ tetra­anion features eight distorted MoO_6_ coordination octa­hedra linked together through bridging O atoms.

## Related literature

For related literature, see: Aguado *et al.* (2005 ▶). 
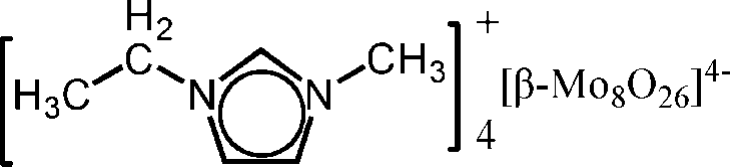


## Experimental

### 

#### Crystal data

(C_6_H_11_N_2_)_4_[Mo_8_O_26_]
*M* *_r_* = 1628.19
Orthorhombic, 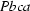

*a* = 15.6338 (6) Å
*b* = 16.9231 (6) Å
*c* = 17.9380 (7) Å
*V* = 4745.9 (3) Å^3^
*Z* = 4
Mo *K*α radiation
μ = 2.13 mm^−1^
*T* = 296 (2) K
0.24 × 0.22 × 0.21 mm

#### Data collection

Bruker APEX CCD area-detector diffractometer
Absorption correction: multi-scan (*SADABS*; Sheldrick, 1996 ▶) *T*_min_ = 0.629, *T*_max_ = 0.663
27619 measured reflections
5677 independent reflections
4568 reflections with *I* > 2σ(*I*)
*R*_int_ = 0.027

#### Refinement

*R*[*F* ^2^ > 2σ(*F* ^2^)] = 0.026
*wR*(*F* ^2^) = 0.070
*S* = 1.04
5677 reflections
298 parameters
H-atom parameters constrained
Δρ_max_ = 0.66 e Å^−3^
Δρ_min_ = −0.61 e Å^−3^

### 

Data collection: *SMART* (Bruker, 1997 ▶); cell refinement: *SAINT* (Bruker, 1999 ▶); data reduction: *SAINT*; program(s) used to solve structure: *SHELXS97* (Sheldrick, 2008 ▶); program(s) used to refine structure: *SHELXL97* (Sheldrick, 2008 ▶); molecular graphics: *SHELXTL-Plus* (Sheldrick, 2008 ▶); software used to prepare material for publication: *SHELXL97*.

## Supplementary Material

Crystal structure: contains datablocks I, global. DOI: 10.1107/S1600536808015936/ya2072sup1.cif

Structure factors: contains datablocks I. DOI: 10.1107/S1600536808015936/ya2072Isup2.hkl

Additional supplementary materials:  crystallographic information; 3D view; checkCIF report

## Figures and Tables

**Fig. 1.**
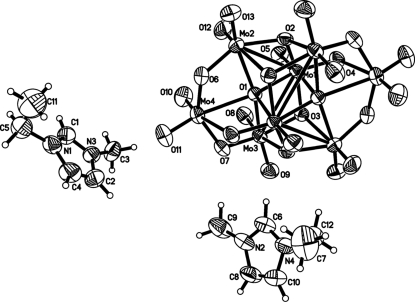
The cations and anion in the structure of the title compound. Thermal displacement ellipsoids are drawn at 30% probability level; H atoms are shown as small circles of arbitrary radius. The unlabeled atoms are related to their symmetry related counterparts by the (2 - x, -y, 1 - z) transformation.

## supplementary crystallographic information

### Comment

The asymmetric unit of the title compound, [emim]_4_[β-Mo_8_O_26_] (emim = 1-ethyl-3-methylimidazolium), (1), contains two [emim]^+^ cations and one half of the [β-Mo_8_O_26_]^4-^ anion, which occupies a special position in the inversion centre (Fig. 1). The anion has eight molybdenum atoms with distorted octahedral coordinations and 26 oxygen atoms which fall into four categories, *i.e.* terminal, µ_2_–, µ_3_ - and µ_5_-bridging atoms. The geometry of tetra-anion is characterized by a wide range of Mo—O distances varying from 1.683 (2) Å for one of the terminal bonds (Mo1—O5) to 2.510 (2) Å for one of the bonds involving 5-coordinated oxygen atom (Mo4—O1). The geometry of the anion is similar to that observed in previous structures, *e.g.* Aguado *et al.* (2005).

### Experimental

A mixture of sodium molydbate, Na_2_MoO_4_.2H_2_O (0.3 mmol), and ionic liquid 1-ethyl-3-methylimidazolium tetrafluoroborate, [emim]BF_4_ (8 ml) was stirred at 170 °C for 24 h in air. The resulting clear solution was filtered and left at room temperature for 3 days. The colourless block crystals were filtered off, washed with cool distilled water and dried in a desiccator at room temperature.

### Refinement

All H-atoms were included in the refinement in a riding model approximation with *U*_iso_=1.2*U*_eq_ (C) for aromatic (C—H 0.93 Å) and methylene (C—H 0.97 Å) H-atoms, *U*_iso_ = 1.5*U*_eq_ (C) for methyl H-atoms (C—H 0.96 Å).

### Crystal data

**Table:**

(C_6_H_11_N_2_)_4_[Mo_8_O_26_]	*F*_000_ = 3152
*M**_r_* = 1628.19	*D*_x_ = 2.279 Mg m^−^^3^
Orthorhombic, *P**b**c**a*	Mo *K*α radiation λ = 0.71073 Å
Hall symbol: -P 2ac 2ab	Cell parameters from 27619 reflections
*a* = 15.6338 (6) Å	θ = 2.1–28.3º
*b* = 16.9231 (6) Å	µ = 2.13 mm^−^^1^
*c* = 17.9380 (7) Å	*T* = 296 (2) K
*V* = 4745.9 (3) Å^3^	Block, colourless
*Z* = 4	0.24 × 0.22 × 0.21 mm

### Data collection

**Table:**

Bruker APEX CCD area-detector diffractometer	5677 independent reflections
Radiation source: fine-focus sealed tube	4568 reflections with *I* > 2σ(*I*)
Monochromator: graphite	*R*_int_ = 0.027
Detector resolution: 0.01 pixels mm^-1^	θ_max_ = 28.3º
*T* = 296(2) K	θ_min_ = 2.1º
ω scans	*h* = −20→14
Absorption correction: multi-scan(SADABS; Sheldrick, 1996)	*k* = −22→22
*T*_min_ = 0.629, *T*_max_ = 0.663	*l* = −20→23
27619 measured reflections	

### Refinement

**Table:**

Refinement on *F*^2^	Secondary atom site location: difference Fourier map
Least-squares matrix: full	Hydrogen site location: inferred from neighbouring sites
*R*[*F*^2^ > 2σ(*F*^2^)] = 0.026	H-atom parameters constrained
*wR*(*F*^2^) = 0.070	*w* = 1/[σ^2^(*F*_o_^2^) + (0.0327*P*)^2^ + 3.2572*P*] where *P* = (*F*_o_^2^ + 2*F*_c_^2^)/3
*S* = 1.04	(Δ/σ)_max_ = 0.002
5677 reflections	Δρ_max_ = 0.66 e Å^−^^3^
298 parameters	Δρ_min_ = −0.61 e Å^−^^3^
Primary atom site location: structure-invariant direct methods	Extinction correction: none

### Special details

**Table:**

Geometry. All e.s.d.'s (except the e.s.d. in the dihedral angle between two l.s. planes) are estimated using the full covariance matrix. The cell e.s.d.'s are taken into account individually in the estimation of e.s.d.'s in distances, angles and torsion angles; correlations between e.s.d.'s in cell parameters are only used when they are defined by crystal symmetry. An approximate (isotropic) treatment of cell e.s.d.'s is used for estimating e.s.d.'s involving l.s. planes.
Refinement. Refinement of *F*^2^ against ALL reflections. The weighted *R*-factor *wR* and goodness of fit *S* are based on *F*^2^, conventional *R*-factors *R* are based on *F*, with *F* set to zero for negative *F*^2^. The threshold expression of *F*^2^ > σ(*F*^2^) is used only for calculating *R*-factors(gt) *etc*. and is not relevant to the choice of reflections for refinement. *R*-factors based on *F*^2^ are statistically about twice as large as those based on *F*, and *R*- factors based on ALL data will be even larger.

### Fractional atomic coordinates and isotropic or equivalent isotropic displacement parameters (Å^2^)

**Table:**

	*x*	*y*	*z*	*U*_iso_*/*U*_eq_	
Mo1	0.919942 (16)	0.001136 (16)	0.429035 (13)	0.03331 (7)	
Mo2	1.078576 (18)	−0.118127 (18)	0.408995 (16)	0.04219 (8)	
Mo3	1.048047 (18)	0.149211 (16)	0.436802 (15)	0.03908 (8)	
Mo4	1.212312 (18)	0.030332 (19)	0.417154 (16)	0.04370 (8)	
O1	1.05538 (13)	0.01203 (12)	0.44123 (11)	0.0345 (4)	
O2	0.95510 (13)	−0.10911 (12)	0.43767 (11)	0.0385 (5)	
O3	0.93026 (12)	0.11030 (12)	0.46027 (12)	0.0372 (5)	
O4	0.81442 (14)	−0.01285 (13)	0.45821 (12)	0.0436 (5)	
O5	0.91133 (15)	0.01019 (14)	0.33592 (12)	0.0490 (6)	
O6	1.19117 (14)	−0.08062 (14)	0.42354 (12)	0.0456 (5)	
O7	1.16753 (14)	0.13133 (13)	0.44644 (13)	0.0470 (5)	
O8	1.03691 (16)	0.15263 (15)	0.34304 (13)	0.0546 (6)	
O9	1.04161 (16)	0.24423 (14)	0.46552 (15)	0.0579 (6)	
O10	1.20274 (17)	0.04091 (16)	0.32313 (14)	0.0617 (7)	
O11	1.31854 (16)	0.03784 (17)	0.43493 (15)	0.0641 (7)	
O12	1.06442 (16)	−0.10397 (17)	0.31615 (14)	0.0631 (7)	
O13	1.09196 (18)	−0.21648 (16)	0.41944 (16)	0.0644 (7)	
N1	1.5874 (2)	0.0109 (2)	0.35763 (17)	0.0562 (8)	
N2	1.2156 (2)	0.28339 (19)	0.6125 (2)	0.0619 (8)	
N3	1.48859 (19)	0.09691 (18)	0.33891 (15)	0.0513 (7)	
N4	1.1552 (2)	0.23736 (18)	0.71109 (19)	0.0599 (8)	
C1	1.5344 (2)	0.0403 (2)	0.3079 (2)	0.0551 (9)	
H1A	1.5299	0.0237	0.2587	0.066*	
C2	1.5147 (3)	0.1051 (3)	0.4115 (2)	0.0647 (11)	
H2A	1.4941	0.1413	0.4461	0.078*	
C3	1.4202 (3)	0.1411 (3)	0.3036 (2)	0.0635 (11)	
H3A	1.4151	0.1251	0.2525	0.095*	
H3B	1.4330	0.1966	0.3058	0.095*	
H3C	1.3674	0.1310	0.3292	0.095*	
C4	1.5752 (3)	0.0513 (3)	0.4230 (2)	0.0702 (12)	
H4A	1.6042	0.0427	0.4675	0.084*	
C5	1.6476 (3)	−0.0559 (3)	0.3444 (2)	0.0754 (13)	
H5A	1.6575	−0.0614	0.2913	0.091*	
H5B	1.7020	−0.0443	0.3680	0.091*	
C6	1.1661 (2)	0.2273 (2)	0.6388 (2)	0.0596 (10)	
H6A	1.1423	0.1866	0.6107	0.072*	
C7	1.1062 (3)	0.1867 (2)	0.7611 (2)	0.0708 (11)	
H7A	1.0702	0.2194	0.7925	0.085*	
H7B	1.0693	0.1528	0.7318	0.085*	
C8	1.2360 (3)	0.3315 (3)	0.6708 (3)	0.0759 (13)	
H8A	1.2698	0.3766	0.6681	0.091*	
C9	1.2417 (3)	0.2920 (3)	0.5351 (3)	0.0872 (15)	
H9A	1.2183	0.2494	0.5062	0.131*	
H9B	1.3030	0.2909	0.5320	0.131*	
H9C	1.2210	0.3414	0.5160	0.131*	
C10	1.1999 (3)	0.3033 (3)	0.7313 (3)	0.0806 (14)	
H10A	1.2041	0.3243	0.7790	0.097*	
C11	1.6139 (4)	−0.1312 (3)	0.3742 (4)	0.115 (2)	
H11A	1.6539	−0.1729	0.3640	0.172*	
H11B	1.5602	−0.1430	0.3508	0.172*	
H11C	1.6059	−0.1265	0.4270	0.172*	
C12	1.1603 (4)	0.1381 (4)	0.8080 (3)	0.118 (2)	
H12A	1.1252	0.1054	0.8392	0.177*	
H12B	1.1955	0.1714	0.8385	0.177*	
H12C	1.1959	0.1054	0.7772	0.177*	

### Atomic displacement parameters (Å^2^)

**Table:**

	*U*^11^	*U*^22^	*U*^33^	*U*^12^	*U*^13^	*U*^23^
Mo1	0.03345 (14)	0.03938 (14)	0.02710 (13)	−0.00539 (11)	−0.00506 (9)	−0.00023 (10)
Mo2	0.04347 (17)	0.04620 (16)	0.03690 (15)	−0.00116 (13)	0.00226 (11)	−0.00868 (12)
Mo3	0.04015 (16)	0.03777 (14)	0.03932 (15)	−0.00623 (12)	−0.00155 (11)	0.00535 (11)
Mo4	0.03562 (15)	0.05413 (18)	0.04136 (16)	−0.00311 (13)	0.00151 (11)	0.00798 (13)
O1	0.0351 (11)	0.0394 (11)	0.0289 (10)	−0.0030 (9)	−0.0017 (8)	0.0008 (8)
O2	0.0416 (12)	0.0382 (11)	0.0357 (11)	−0.0072 (9)	−0.0048 (9)	−0.0064 (9)
O3	0.0364 (11)	0.0367 (11)	0.0385 (12)	0.0003 (9)	−0.0014 (9)	0.0001 (9)
O4	0.0362 (12)	0.0553 (13)	0.0392 (12)	−0.0066 (10)	−0.0057 (9)	0.0017 (10)
O5	0.0548 (14)	0.0600 (15)	0.0322 (12)	−0.0031 (11)	−0.0100 (10)	0.0004 (10)
O6	0.0393 (12)	0.0519 (13)	0.0456 (13)	0.0050 (11)	0.0061 (9)	0.0019 (10)
O7	0.0383 (12)	0.0488 (13)	0.0540 (14)	−0.0123 (10)	−0.0017 (10)	0.0065 (11)
O8	0.0538 (15)	0.0651 (16)	0.0450 (14)	−0.0102 (12)	−0.0027 (11)	0.0139 (12)
O9	0.0633 (16)	0.0413 (13)	0.0689 (17)	−0.0040 (12)	−0.0009 (13)	−0.0003 (12)
O10	0.0706 (18)	0.0699 (17)	0.0446 (14)	0.0031 (14)	0.0051 (12)	0.0117 (12)
O11	0.0398 (14)	0.083 (2)	0.0689 (17)	−0.0065 (13)	0.0012 (12)	0.0155 (14)
O12	0.0624 (17)	0.089 (2)	0.0376 (13)	−0.0027 (14)	0.0031 (11)	−0.0157 (13)
O13	0.0703 (18)	0.0481 (15)	0.0748 (19)	0.0002 (13)	0.0033 (14)	−0.0174 (13)
N1	0.0514 (18)	0.072 (2)	0.0457 (17)	−0.0034 (15)	−0.0109 (14)	−0.0023 (15)
N2	0.0543 (19)	0.0583 (19)	0.073 (2)	−0.0168 (16)	−0.0121 (16)	0.0017 (17)
N3	0.0540 (18)	0.0637 (18)	0.0363 (15)	−0.0049 (15)	0.0002 (13)	−0.0049 (14)
N4	0.0528 (18)	0.0563 (18)	0.070 (2)	−0.0094 (15)	−0.0104 (16)	−0.0100 (16)
C1	0.055 (2)	0.072 (2)	0.0383 (18)	0.0020 (19)	−0.0073 (16)	−0.0061 (17)
C2	0.069 (3)	0.085 (3)	0.041 (2)	−0.002 (2)	0.0005 (18)	−0.013 (2)
C3	0.063 (3)	0.078 (3)	0.049 (2)	0.016 (2)	0.0034 (18)	−0.004 (2)
C4	0.077 (3)	0.098 (4)	0.036 (2)	−0.010 (3)	−0.0116 (18)	−0.004 (2)
C5	0.064 (3)	0.097 (3)	0.065 (3)	0.016 (3)	−0.024 (2)	−0.011 (2)
C6	0.061 (2)	0.055 (2)	0.064 (2)	−0.0145 (19)	−0.0155 (19)	−0.0003 (19)
C7	0.063 (3)	0.073 (3)	0.076 (3)	−0.004 (2)	−0.004 (2)	−0.004 (2)
C8	0.069 (3)	0.058 (2)	0.100 (4)	−0.023 (2)	−0.006 (3)	−0.017 (2)
C9	0.088 (3)	0.097 (4)	0.077 (3)	−0.038 (3)	−0.011 (3)	0.015 (3)
C10	0.076 (3)	0.079 (3)	0.087 (3)	−0.017 (2)	−0.003 (3)	−0.033 (3)
C11	0.124 (5)	0.087 (4)	0.134 (5)	0.016 (4)	−0.007 (4)	0.019 (4)
C12	0.099 (4)	0.143 (5)	0.112 (5)	0.022 (4)	−0.008 (4)	0.047 (4)

### Geometric parameters (Å, °)

**Table:**

Mo1—O5	1.683 (2)	N2—C8	1.363 (5)
Mo1—O4	1.747 (2)	N2—C9	1.454 (6)
Mo1—O3	1.937 (2)	N3—C1	1.319 (5)
Mo1—O2	1.951 (2)	N3—C2	1.372 (4)
Mo1—O1	2.137 (2)	N3—C3	1.451 (5)
Mo1—O1^i^	2.370 (2)	N4—C6	1.319 (5)
Mo1—Mo3	3.2109 (4)	N4—C10	1.366 (5)
Mo1—Mo2	3.2177 (4)	N4—C7	1.458 (5)
Mo2—O13	1.688 (3)	C1—H1A	0.9300
Mo2—O12	1.697 (3)	C2—C4	1.328 (6)
Mo2—O6	1.889 (2)	C2—H2A	0.9300
Mo2—O2	2.004 (2)	C3—H3A	0.9600
Mo2—O1	2.306 (2)	C3—H3B	0.9600
Mo2—O3^i^	2.353 (2)	C3—H3C	0.9600
Mo3—O9	1.692 (2)	C4—H4A	0.9300
Mo3—O8	1.692 (2)	C5—C11	1.478 (7)
Mo3—O7	1.900 (2)	C5—H5A	0.9700
Mo3—O3	2.000 (2)	C5—H5B	0.9700
Mo3—O1	2.326 (2)	C6—H6A	0.9300
Mo3—O2^i^	2.352 (2)	C7—C12	1.448 (6)
Mo4—O11	1.696 (3)	C7—H7A	0.9700
Mo4—O10	1.703 (2)	C7—H7B	0.9700
Mo4—O6	1.910 (2)	C8—C10	1.313 (6)
Mo4—O7	1.920 (2)	C8—H8A	0.9300
Mo4—O4^i^	2.294 (2)	C9—H9A	0.9600
Mo4—O1	2.510 (2)	C9—H9B	0.9600
O1—Mo1^i^	2.370 (2)	C9—H9C	0.9600
O2—Mo3^i^	2.352 (2)	C10—H10A	0.9300
O3—Mo2^i^	2.353 (2)	C11—H11A	0.9600
O4—Mo4^i^	2.294 (2)	C11—H11B	0.9600
N1—C1	1.315 (4)	C11—H11C	0.9600
N1—C4	1.371 (5)	C12—H12A	0.9600
N1—C5	1.490 (5)	C12—H12B	0.9600
N2—C6	1.313 (5)	C12—H12C	0.9600
			
O5—Mo1—O4	103.54 (11)	Mo1—O1—Mo2	92.74 (7)
O5—Mo1—O3	101.94 (10)	Mo1—O1—Mo3	91.94 (8)
O4—Mo1—O3	96.96 (9)	Mo2—O1—Mo3	162.30 (10)
O5—Mo1—O2	100.86 (10)	Mo1—O1—Mo1^i^	104.70 (8)
O4—Mo1—O2	96.47 (10)	Mo2—O1—Mo1^i^	97.52 (7)
O3—Mo1—O2	149.93 (8)	Mo3—O1—Mo1^i^	97.78 (7)
O5—Mo1—O1	99.96 (10)	Mo1—O1—Mo4	164.07 (10)
O4—Mo1—O1	156.48 (9)	Mo2—O1—Mo4	85.47 (7)
O3—Mo1—O1	78.79 (8)	Mo3—O1—Mo4	85.36 (6)
O2—Mo1—O1	78.18 (8)	Mo1^i^—O1—Mo4	91.23 (7)
O5—Mo1—O1^i^	175.21 (10)	Mo1—O2—Mo2	108.90 (10)
O4—Mo1—O1^i^	81.19 (8)	Mo1—O2—Mo3^i^	110.24 (9)
O3—Mo1—O1^i^	78.00 (8)	Mo2—O2—Mo3^i^	104.12 (9)
O2—Mo1—O1^i^	77.66 (8)	Mo1—O3—Mo3	109.25 (10)
O1—Mo1—O1^i^	75.30 (8)	Mo1—O3—Mo2^i^	109.70 (9)
O5—Mo1—Mo3	91.25 (8)	Mo3—O3—Mo2^i^	104.20 (9)
O4—Mo1—Mo3	132.98 (7)	Mo1—O4—Mo4^i^	118.79 (10)
O3—Mo1—Mo3	36.03 (6)	Mo2—O6—Mo4	118.90 (12)
O2—Mo1—Mo3	124.55 (6)	Mo3—O7—Mo4	118.38 (11)
O1—Mo1—Mo3	46.38 (5)	C1—N1—C4	107.6 (4)
O1^i^—Mo1—Mo3	85.94 (5)	C1—N1—C5	125.2 (3)
O5—Mo1—Mo2	90.45 (8)	C4—N1—C5	127.1 (3)
O4—Mo1—Mo2	132.56 (7)	C6—N2—C8	107.1 (4)
O3—Mo1—Mo2	124.49 (6)	C6—N2—C9	125.5 (3)
O2—Mo1—Mo2	36.09 (6)	C8—N2—C9	127.4 (4)
O1—Mo1—Mo2	45.71 (5)	C1—N3—C2	108.1 (3)
O1^i^—Mo1—Mo2	85.71 (5)	C1—N3—C3	126.2 (3)
Mo3—Mo1—Mo2	90.780 (10)	C2—N3—C3	125.6 (3)
O13—Mo2—O12	105.32 (14)	C6—N4—C10	107.5 (4)
O13—Mo2—O6	101.56 (12)	C6—N4—C7	126.6 (3)
O12—Mo2—O6	102.10 (11)	C10—N4—C7	125.9 (4)
O13—Mo2—O2	99.56 (11)	N1—C1—N3	109.4 (3)
O12—Mo2—O2	96.63 (10)	N1—C1—H1A	125.3
O6—Mo2—O2	146.78 (9)	N3—C1—H1A	125.3
O13—Mo2—O1	159.00 (11)	C4—C2—N3	106.9 (4)
O12—Mo2—O1	95.20 (11)	C4—C2—H2A	126.6
O6—Mo2—O1	77.94 (9)	N3—C2—H2A	126.6
O2—Mo2—O1	73.22 (8)	N3—C3—H3A	109.5
O13—Mo2—O3^i^	87.26 (11)	N3—C3—H3B	109.5
O12—Mo2—O3^i^	164.26 (11)	H3A—C3—H3B	109.5
O6—Mo2—O3^i^	84.15 (8)	N3—C3—H3C	109.5
O2—Mo2—O3^i^	71.54 (7)	H3A—C3—H3C	109.5
O1—Mo2—O3^i^	71.77 (7)	H3B—C3—H3C	109.5
O13—Mo2—Mo1	134.56 (10)	C2—C4—N1	108.0 (3)
O12—Mo2—Mo1	85.45 (9)	C2—C4—H4A	126.0
O6—Mo2—Mo1	119.47 (7)	N1—C4—H4A	126.0
O2—Mo2—Mo1	35.01 (6)	C11—C5—N1	111.8 (4)
O1—Mo2—Mo1	41.55 (5)	C11—C5—H5A	109.3
O3^i^—Mo2—Mo1	78.94 (5)	N1—C5—H5A	109.3
O9—Mo3—O8	105.31 (13)	C11—C5—H5B	109.3
O9—Mo3—O7	100.50 (11)	N1—C5—H5B	109.3
O8—Mo3—O7	101.36 (11)	H5A—C5—H5B	107.9
O9—Mo3—O3	101.19 (11)	N2—C6—N4	109.6 (3)
O8—Mo3—O3	97.23 (10)	N2—C6—H6A	125.2
O7—Mo3—O3	146.45 (9)	N4—C6—H6A	125.2
O9—Mo3—O1	160.30 (10)	C12—C7—N4	112.6 (4)
O8—Mo3—O1	94.20 (10)	C12—C7—H7A	109.1
O7—Mo3—O1	77.85 (8)	N4—C7—H7A	109.1
O3—Mo3—O1	73.12 (8)	C12—C7—H7B	109.1
O9—Mo3—O2^i^	88.94 (10)	N4—C7—H7B	109.1
O8—Mo3—O2^i^	163.60 (10)	H7A—C7—H7B	107.8
O7—Mo3—O2^i^	83.54 (9)	C10—C8—N2	108.5 (4)
O3—Mo3—O2^i^	71.60 (8)	C10—C8—H8A	125.8
O1—Mo3—O2^i^	71.36 (7)	N2—C8—H8A	125.8
O9—Mo3—Mo1	135.89 (9)	N2—C9—H9A	109.5
O8—Mo3—Mo1	85.38 (8)	N2—C9—H9B	109.5
O7—Mo3—Mo1	119.53 (7)	H9A—C9—H9B	109.5
O3—Mo3—Mo1	34.72 (6)	N2—C9—H9C	109.5
O1—Mo3—Mo1	41.69 (5)	H9A—C9—H9C	109.5
O2^i^—Mo3—Mo1	78.66 (5)	H9B—C9—H9C	109.5
O11—Mo4—O10	105.33 (13)	C8—C10—N4	107.3 (4)
O11—Mo4—O6	103.41 (12)	C8—C10—H10A	126.3
O10—Mo4—O6	98.49 (11)	N4—C10—H10A	126.3
O11—Mo4—O7	103.82 (12)	C5—C11—H11A	109.5
O10—Mo4—O7	98.36 (11)	C5—C11—H11B	109.5
O6—Mo4—O7	142.70 (9)	H11A—C11—H11B	109.5
O11—Mo4—O4^i^	90.28 (10)	C5—C11—H11C	109.5
O10—Mo4—O4^i^	164.39 (11)	H11A—C11—H11C	109.5
O6—Mo4—O4^i^	77.48 (8)	H11B—C11—H11C	109.5
O7—Mo4—O4^i^	77.39 (9)	C7—C12—H12A	109.5
O11—Mo4—O1	159.07 (10)	C7—C12—H12B	109.5
O10—Mo4—O1	95.59 (10)	H12A—C12—H12B	109.5
O6—Mo4—O1	72.50 (8)	C7—C12—H12C	109.5
O7—Mo4—O1	72.93 (8)	H12A—C12—H12C	109.5
O4^i^—Mo4—O1	68.80 (7)	H12B—C12—H12C	109.5

Symmetry codes: (i) −*x*+2, −*y*, −*z*+1.
